# Ultrasound viscosity imaging empowers BI-RADS: toward precise breast lesion diagnosis and analysis of HER2 status

**DOI:** 10.3389/fonc.2026.1726418

**Published:** 2026-02-25

**Authors:** Yiming Chen, Jialing Wu, Yiting Liu, Xiukun Hou

**Affiliations:** Department of Ultrasound I, the First Affiliated Hospital of Dalian Medical University, Dalian, Liaoning, China

**Keywords:** BI-RADS, breast lesions, HER2, ultrasound, viscosity

## Abstract

**Introduction:**

Breast cancer remains a major challenge in women’s health globally. Early screening and personalized treatment can improve outcomes. This study aimed to evaluate ultrasound viscosity imaging (UVI) for distinguishing benign from malignant breast lesions and noninvasively assessing human epidermal growth factor receptor 2 (HER2) status.

**Materials and methods:**

We conducted a retrospective analysis of 274 breast lesions, randomly divided into a derivation cohort and a validation cohort (VC) at a 7:3 ratio. Breast Imaging Reporting and Data System (BI-RADS) scores and UVI parameters were collected, with histopathology as the reference standard. The Boruta algorithm was used to identify the optimal viscous parameter (VP). A logistic regression model assessed the diagnostic performance of BI-RADS alone and in combination with VP. Associations between viscous parameters (VPs) and HER2 status were also examined.

**Results:**

Among 40 VPs, V2.max (maximum viscosity from the Voigt model within a perilesional 2-mm rim) was identified as the optimal marker. When combined with BI-RADS, V2.max enhanced the differentiation between benign and malignant lesions (p<0.001), increasing the area under the curve (AUC) from 0.91 (95% CI: 0.87-0.95) to 0.96 (95% CI: 0.94-0.98). The combined model also demonstrated superior calibration, which was revalidated in the VC. Subgroup analyses confirmed its effectiveness in younger patients and those with larger lesions. Furthermore, we identified four Voigt-model-derived VPs, including V2.max, that correlated with HER2 positivity, and explored their potential histological basis.

**Conclusion:**

UVI-derived VPs enhance BI-RADS diagnostic performance for breast lesions and are associated with HER2 status.

## 1 Introduction

Breast cancer (BC) poses a significant global health burden, accounting for a substantial portion of cancer diagnoses in women annually (1). Early screening and timely treatment of high-risk populations are therefore important strategies to improve patient outcomes and reduce the global burden of this disease (2). While tissue biopsy remains the gold standard for distinguishing benign from malignant breast lesions, its invasive nature and associated discomfort make it unsuitable for large-scale screening (3). The current mainstream screening modality, mammography, also faces significant limitations, including constrained medical resources and significantly reduced sensitivity in dense breasts (4, 5). As a complement to mammography, sonomammography has demonstrated considerable clinical value (6). Among these, ultrasound elastography is recommended as an adjunct to the Breast Imaging Reporting and Data System (BI-RADS) to enhance overall diagnostic performance, as it significantly enhances the sensitivity and specificity of conventional ultrasound (7).Ultrasound elastography is based on the assumption that biological tissue behaves as a purely elastic solid, inferring tissue elasticity indirectly by measuring shear wave velocity (8). In reality, however, biological tissues exhibit viscoelastic properties, whereby the velocity of shear wave propagation increases with frequency—a phenomenon known as dispersion. This inherent property inevitably introduces deviations in clinical practice (9). The development of ultrasound viscosity imaging (UVI) has addressed this limitation. By acquiring tissue shear wave velocities across multiple frequencies and fitting them to rheological models like the Voigt model, UVI enables the calculation of quantitative viscous parameters (VPs). This provides a more comprehensive evaluation of tissue biomechanical properties (8). Multiple studies have demonstrated that VPs are closely associated with liver fibrosis and inflammatory responses (10, 11), it also exhibits potential value in the diagnosis and monitoring of chronic kidney disease (12, 13). These findings highlight the clinical potential of UVI in evaluating parenchymal organ pathology.

To date, only a limited number of studies have investigated the value of UVI in differentiating benign from malignant breast lesions (8, 14, 15). While Kumar et al. pioneered Voigt model-based viscosity imaging in small samples (8) and Bae et al. later validated UVI feasibility in a larger cohort using the SWD model (15), both studies highlighted the need for further development. Building on this, the multicenter study by Jia et al. provided a systematic analysis of UVI. They innovatively utilized the selected optimal viscous parameter (VP) as an adjunctive score to BI-RADS to adjust its category, demonstrating the potential of UVI and providing significant clinical guidance (14). However, this study has limitations. Although the area under the curve (AUC) of the modified BI-RADS significantly increased from 0.85 to 0.90 (*p* < 0.05) in the overall cohort, Jia et al. did not develop a novel, interpretable diagnostic model. Furthermore, no study to date has analyzed the correlation between Human epidermal growth factor receptor 2 (HER2) status and VPs. Therefore, further investigation into the value of UVI for evaluating breast lesions is warranted. This study aimed to integrate the optimal VP with BI-RADS using logistic regression, to develop and evaluate a combined model for differentiating breast lesions and to validate the clinical utility of UVI more robustly. Additionally, we explored the correlation between VPs and HER2 status to investigate the potential of UVI for the non-invasive assessment of this crucial therapeutic target.

## 2 Materials and methods

### 2.1 Research subjects

This retrospective study was conducted in accordance with the Declaration of Helsinki and was approved by the Institutional Review Board of the author’s institution (Approval No. PJ-KS-KY-2025-444; Date: May 20, 2025). Owing to the retrospective design, the requirement for informed consent was waived by the Institutional Review Board. Between December 2024 and May 2025, a total of 258 female patients with breast diseases who underwent both conventional sonomammography and UVI at our hospital and subsequently received core needle biopsy or surgery with available histopathological results, were included in this study.

**Figure 1:**
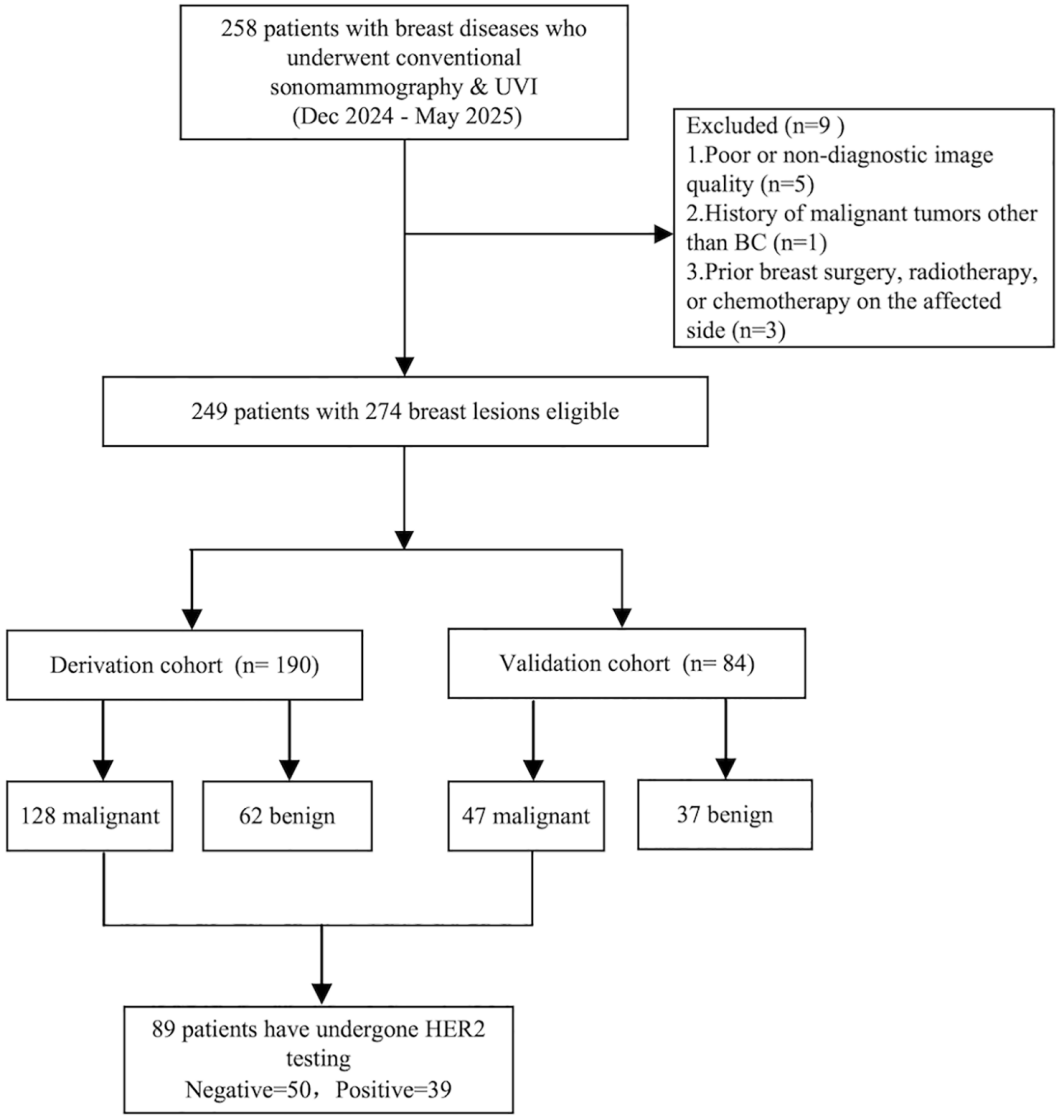
Study flowchart. The diagram illustrates the patient selection process, application of exclusion criteria, and final cohort allocation for the 274 breast lesions included in the study. DC, derivation cohort; VC, validation cohort; HER2, human epidermal growth factor receptor 2; IHC, immunohistochemistry; FISH, fluorescence *in situ* hybridization.

Patient selection followed predefined criteria. The inclusion criteria were: (1) presence of diagnostic-quality conventional ultrasound and UVI images; (2) definitive histopathological confirmation (from biopsy or surgical specimens); (3) non-pregnant and non-lactating status; and (4) presence of at least one ultrasound-detectable breast lesion. The exclusion criteria were: (1) poor or non-diagnostic image quality (n = 5); (2) history of malignant tumors other than BC (n = 1); and (3) prior breast surgery, radiotherapy, or chemotherapy on the affected side (n = 3). Ultimately, the study included 274 breast lesions from 249 patients (21 with 2 lesions, 2 with 3 lesions). Of these, 89 lesions underwent HER2 testing. All lesions were randomly split 7:3 into derivation (DC, n=190) and validation (VC, n=84) cohorts. HER2-tested lesions were stratified by immunohistochemistry (IHC)/fluorescence *in situ* hybridization (FISH) results into positive (n=39) and negative (n=50) groups (**Figure 1**).

### 2.2 Image acquisition and analysis

**Figure 2:**
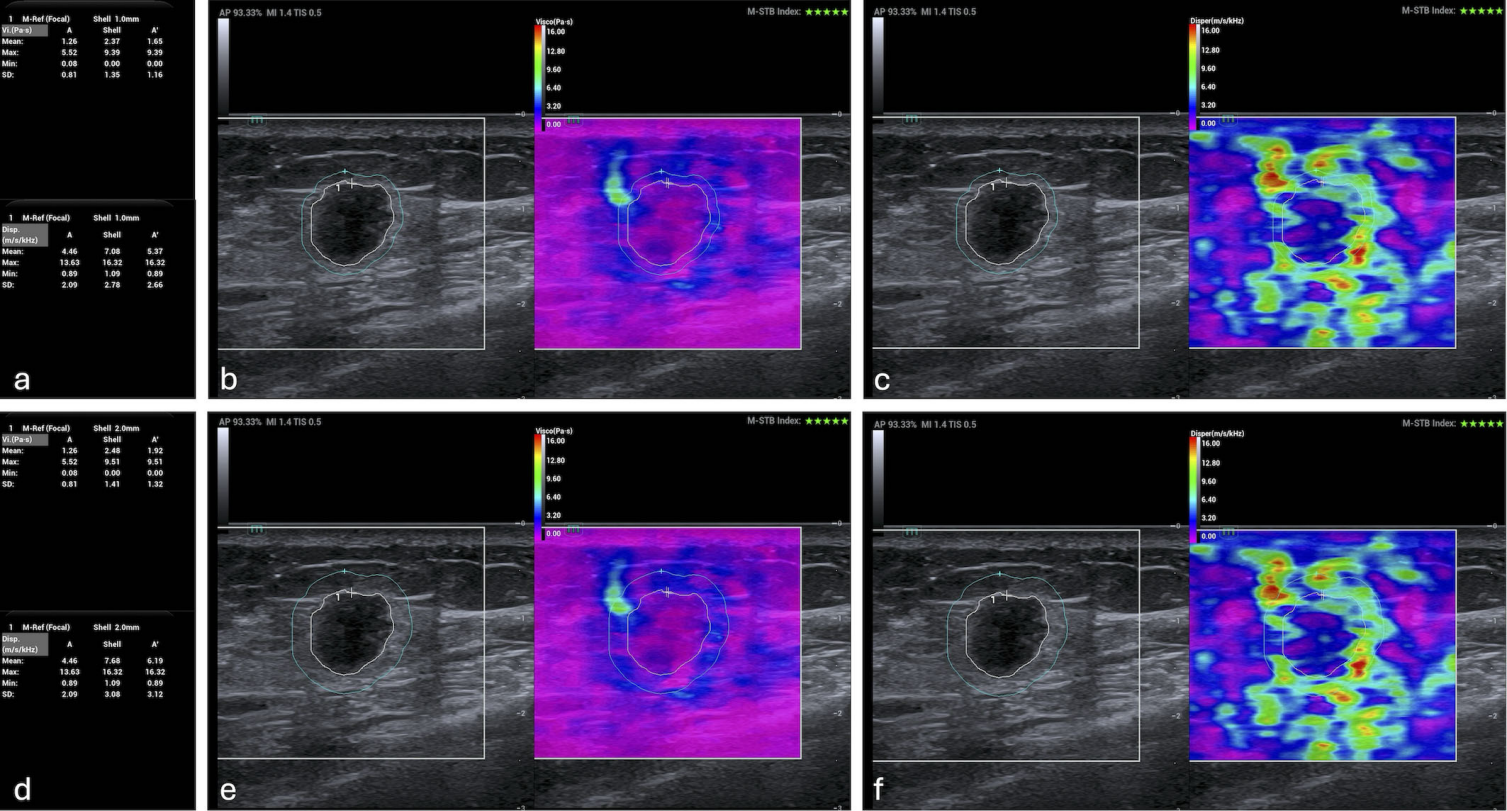
Representative ultrasound viscosity imaging analysis of a breast lesion. **(a, d)** Quantitative viscous parameter (VP) maps derived from the Voigt (units: Pa·s) and shear wave dispersion (SWD) (units: m/s/kHz) models, respectively. For each model, four statistical parameters (maximum, minimum, mean, and standard deviation) are displayed for three regions of interest: the lesion core (A), a 1-mm perilesional rim (Shell 1), and a 2-mm perilesional rim (Shell 2). **(b, c, e, f)** The corresponding grayscale ultrasound images underlaid with the VP distributions shown in panels a and d.

Two sonographers, each possessing over a decade of experience in sonomammography and specialized training in standardized UVI operation, performed the breast lesion image acquisition. Both sonographers were blinded to all clinical and other imaging findings of the examined patients. All images were acquired using a Mindray Resona A20s ultrasound system (Shenzhen Mindray Bio-Medical Electronics Co. Ltd, Shenzhen, China) equipped with an LM18-5WU linear array transducer operating at a frequency range of 5–18 MHz. The system provides two quality indices—Motion Stability and Reliability—to evaluate image adequacy. Before imaging, patients were instructed to assume a supine position with both upper limbs elevated to fully expose the axillary and breast regions. Conventional ultrasound imaging was then performed, and a quantitative BI-RADS score was assigned to each lesion based on its characteristic features, such as size, morphology, margin, and the presence of calcifications. Next, the optimal cross-section of the target lesion was selected in grayscale mode, after which the system was switched to UVI mode and the sampling box was adjusted. A region of interest was defined, and patients were instructed to hold their breath. The probe was placed gently on the skin surface without compression and held stable for 3 s. Images were frozen and stored only if they satisfied the following criteria: (1) Motion Stability index ≥4 stars and (2) Reliability index ≥95%. Upon acquiring satisfactory images, the lesion contour was delineated on the grayscale ultrasound image using the system’s built-in Shell software package. The Shell value was adjusted to one or two to define the lesion core and the surrounding 1-mm and 2-mm perilesional rims for analysis. VPs were then derived from these regions based on both the Voigt model and the SWD model. **Table 1** summarizes the specific acquisition parameters, which are visually presented in **Figure 2**. All VP values reported represent the mean of three consecutive measurements.

**Table 1:** Nomenclature of viscosity parameters.

	Voigt Model				SWD Model			
Parameter	Max	Min	Mean	Sd	Max	Min	Mean	Sd
Lesion Core	Vmax	Vmin	Vmean	Vsd	Dmax	Dmin	Dmean	Dsd
Perilesional, 1-mm	V1.max	V1.min	V1.mean	V1.sd	D1.max	D1.min	D1.mean	D1.sd
Perilesional, 1-mm	V2.max	V2.min	V2.mean	V2.sd	D2.max	D2.min	D2.mean	D2.sd
Core + 1-mmPL	A’V1.max	A’V1.min	A’V1.mean	A’V1.sd	A’D1.max	A’D1.min	A’D1.mean	A’D1.sd
Core + 2-mmPL	A’V2.max	A’V2.min	A’V2.mean	A’V2.sd	A’D2.max	A’D2.min	A’D2.mean	A’D2.sd

This table defines the naming convention for all viscous parameters (VPs) derived from the Voigt and shear wave dispersion (SWD) models. The prefix indicates the region of interest (e.g., V for lesion core; V1 for 1-mm perilesional rim; A’V1 for combined core and 1-mm rim), and the suffix indicates the statistical metric (max, min, mean, sd).

### 2.3 Histopathological evaluation

Histopathological reports from core needle biopsies and surgical excisions for all enrolled patients were collected. These reports included the pathological diagnosis and HER2 status results obtained from both IHC and FISH. Lesions were categorized into benign or malignant based on the pathological diagnosis. For HER2 assessment, an IHC score of three was defined as HER2 positive, whereas scores of zero or one were considered negative. Cases with an IHC score of two required additional evaluation with FISH (16). Two board-certified pathologists, each possessing over a decade of specialized experience in breast histopathology, independently performed all procedures. To mitigate potential bias, neither pathologist had access to the clinical or imaging data.

### 2.4 Subgroup analysis

Lesion size and patient age, as fundamental parameters in disease assessment, play a pivotal role in clinical management and therapeutic decision-making (17–20). Previous studies have commonly used a maximum lesion diameter of ≤20 mm as the cut-off value for defining breast lesion size (21, 22), although the stratification of age groups has lacked consensus (19, 23). In this study, lesions with a maximum diameter of ≤20 mm were defined as small lesions, and patients aged ≤45 years were categorized as the young group. Based on these criteria, the diagnostic performance of the model was evaluated across subgroups stratified by lesion size and patient age to assess its applicability in specific patient populations.

### 2.5 Statistical analysis

Data were expressed as mean ± standard deviation for normally distributed continuous variables, median (interquartile range [IQR]) for non-normally distributed variables, and frequency (percentage) for categorical variables. Group comparisons (benign vs. malignant; HER2 positive vs. negative) were performed as follows: continuous variables were assessed using Student’s t-test or the Wilcoxon rank-sum test, while categorical variables were evaluated with the chi-square (χ²) test or Fisher’s exact test. Variance inflation factors were calculated for all VPs to assess multicollinearity. The optimal VP was selected by applying the Boruta algorithm (R Boruta package, version 8.0.0; maxRuns= 100 iterations) exclusively to the derivation cohort, preventing information leakage. Two binary logistic regression models were subsequently constructed: the original BI-RADS model (BI-RADS-O) and the BI-RADS plus optimal VP model (referred to here as the viscosity-modified BI-RADS model, BI-RADS-V). Model performance was evaluated across multiple domains: discrimination (AUC), calibration (calibration curves, Brier score, the Hosmer–Lemeshow [H-L] test), reclassification (net reclassification improvement [NRI], integrated discrimination improvement [IDI]), and clinical utility (decision curve analysis [DCA]). Sensitivity and specificity were also assessed. To evaluate the impact of within-patient lesion clustering on the BI-RADS-V model estimates in the DC, a sensitivity analysis using Generalized Estimating Equations with an exchangeable correlation structure was conducted, with the intraclass correlation coefficient (ICC) used for quantification. After completing all analyses in the DC, the generalizability of the BI-RADS-V was validated in the VC. Breast lesion size and patient age were also used as grouping variables to evaluate the applicability of BI-RADS-V across patient subgroups within the DC. Finally, a univariable logistic regression analysis was performed for all VPs to explore their association with HER2 status. All statistical analyses were performed using R software, version 4.5.1 (R Foundation for Statistical Computing, Vienna, Austria).

### 2.6 Reproducibility analysis

To assess the intra-observer reproducibility of the key ultrasound viscosity parameter, we utilized the three consecutive measurements obtained for each lesion. The ICC was calculated using a two-way random-effects model for absolute agreement based on single measurements (ICC).

## 3 Result

### 3.1 Demographic characteristics

In both the DC and VC cohorts, **Table 2** presents comparisons of age, conventional ultrasound parameters, and VPs between the benign and malignant groups. The malignant group demonstrated significantly higher values for age, lesion diameter, and BI-RADS score (*p* < 0.05). In the DC, all VPs except V1.mean, A’V1.mean, A’V2.mean, Dmin, D1.min, D2.min, A’D1.min, and A’D2.min showed statistically significant differences (*p* < 0.05). Conversely, statistically significant differences (*p* < 0.05) in the VC were shown by only 21 VPs, including Vmax.

**Table 2:** Baseline characteristics of patients and lesions.

Parameter	Derivation cohort (n=190)			Validation cohort (n=84)		
	Benign (n=62)	Malignant (n=128)	P value	Benign (n=37)	Malignant (n=47)	P value
Age, year	47.50 (41.25-58.75)	60.00 (50.00-68.25)	<0.001	44.00 (42.00-48.00)	60.00 (53.00-66.00)	<0.001
BI-RADS	7.00 (5.00-8.00)	12.00 (9.00-13.25)	<0.001	6.00 (6.00-7.00)	10.00 (9.00-13.00)	<0.001
Diameter, mm	14.50 (10.00-19.00)	20.00 (15.75-29.00)	<0.001	13.00 (9.00-23.00)	20.00 (12.50-26.00)	0.014
Voigt parameters, Pa.s						
Vmax	3.00 (2.43-3.90)	4.78 (3.55-5.68)	<0.001	3.29 (2.74-5.01)	4.59 (3.54-6.44)	0.002
Vmin	0.00 (0.00-0.15)	0.00 (0.00-0.00)	0.004	0.00 (0.00-0.08)	0.00 (0.00-0.02)	0.437
Vmean	1.02 (0.86-1.38)	0.90 (0.64-1.29)	0.011	1.17 (1.01-1.56)	0.99 (0.70-1.46)	0.092
Vsd	0.52 (0.41-0.66)	0.66 (0.51-0.81)	<0.001	0.64 (0.49-0.82)	0.66 (0.49-1.02)	0.471
V1.max	3.10 (2.37-3.98)	6.05 (4.44-7.57)	<0.001	3.27 (2.92-5.24)	6.25 (5.00-8.45)	<0.001
V1.min	0.01 (0.00-0.11)	0.00 (0.00-0.03)	0.002	0.01 (0.00-0.14)	0.00 (0.00-0.07)	0.506
V1.mean	1.07 (0.89-1.44)	1.27 (0.95-1.64)	0.062	1.40 (1.03-1.80)	1.36 (1.09-1.69)	0.896
V1.sd	0.57 (0.46-0.77)	1.02 (0.77-1.25)	<0.001	0.71 (0.56-1.05)	0.99 (0.77-1.45)	0.001
V2.max	3.34 (2.58-4.35)	6.96 (5.28-8.24)	<0.001	3.60 (3.14-5.81)	6.74 (5.54-10.19)	<0.001
V2.min	0.00 (0.00-0.07)	0.00 (0.00-0.00)	0.006	0.00 (0.00-0.10)	0.00 (0.00-0.03)	0.354
V2.mean	1.08 (0.89-1.40)	1.33 (0.96-1.64)	0.010	1.30 (1.06-1.63)	1.38 (1.17-1.73)	0.340
V2.sd	0.63 (0.49-0.77)	1.07 (0.79-1.27)	<0.001	0.74 (0.58-0.95)	1.16 (0.85-1.48)	<0.001
A’V1.max	3.31 (2.46-4.39)	6.05 (4.58-7.78)	<0.001	3.56 (3.04-5.61)	6.35 (5.03-8.55)	<0.001
A’V1.min	0.00 (0.00-0.05)	0.00 (0.00-0.00)	0.005	0.00 (0.00-0.04)	0.00 (0.00-0.01)	0.521
A’V1.mean	1.07 (0.88-1.45)	1.02 (0.72-1.43)	0.126	1.27 (1.05-1.53)	1.06 (0.79-1.50)	0.143
A’V1.sd	0.57 (0.45-0.69)	0.81 (0.59-0.96)	<0.001	0.65 (0.55-0.96)	0.79 (0.64-1.17)	0.128
A’V2.max	3.51 (2.70-4.62)	6.97 (5.37-8.36)	<0.001	3.82 (3.20-5.93)	6.74 (5.75-10.19)	<0.001
A’V2.min	0.00 (0.00-0.05)	0.00 (0.00-0.00)	0.006	0.00 (0.00-0.03)	0.00 (0.00-0.01)	0.505
A’V2.mean	1.06 (0.87-1.40)	1.10 (0.79-1.46)	0.626	1.26 (1.07-1.53)	1.15 (0.90-1.52)	0.337
A’V2.sd	0.59 (0.47-0.72)	0.90 (0.66-1.09)	<0.001	0.67 (0.57-0.99)	0.93 (0.71-1.20)	0.022
SWD parameters, m/s/kHz						
Dmax	11.76 (8.26-15.07)	15.81 (13.77-18.95)	<0.001	14.12 (12.25-16.99)	17.28 (15.51-19.40)	<0.001
Dmin	0.06 (0.00-0.70)	0.13 (0.00-0.63)	0.411	0.23 (0.00-0.85)	0.39 (0.00-0.71)	0.840
Dmean	4.35 (3.23-5.28)	4.85 (3.94-6.23)	0.003	4.96 (4.14-6.12)	5.34 (4.63-7.18)	0.241
Dsd	2.18 (1.70-2.54)	2.72 (2.04-3.24)	<0.001	2.62 (2.27-2.92)	2.90 (2.43-3.31)	0.062
D1.max	12.29 (9.10-14.62)	16.41 (14.19-19.32)	<0.001	13.88 (11.81-16.16)	17.93 (15.83-19.86)	<0.001
D1.min	0.18 (0.00-0.74)	0.22 (0.00-0.72)	0.961	0.36 (0.00-0.81)	0.41 (0.00-1.07)	0.410
D1.mean	4.51 (3.30-5.41)	5.69 (4.77-7.21)	<0.001	5.08 (4.20-6.12)	6.42 (5.40-7.55)	<0.001
D1.sd	2.53 (1.88-2.99)	3.20 (2.63-3.73)	<0.001	2.94 (2.23-3.42)	3.35 (2.96-3.92)	0.002
D2.max	13.04 (10.01-14.98)	17.30 (15.02-19.92)	<0.001	14.37 (12.00-17.34)	18.15 (16.21-19.86)	<0.001
D2.min	0.04 (0.00-0.32)	0.03 (0.00-0.39)	0.748	0.17 (0.00-0.56)	0.15 (0.00-0.77)	0.708
D2.mean	4.25 (3.23-5.24)	5.50 (4.76-7.04)	<0.001	4.88 (3.93-5.74)	6.38 (5.45-7.33)	<0.001
D2.sd	2.44 (1.87-2.97)	3.20 (2.68-3.75)	<0.001	2.87 (2.16-3.32)	3.44 (3.03-4.05)	<0.001
A’D1.max	12.50 (10.02-15.53)	16.66 (15.23-19.95)	<0.001	14.75 (12.47-17.02)	18.52 (16.38-19.94)	<0.001
A’D1.min	0.01 (0.00-0.48)	0.04 (0.00-0.42)	0.559	0.06 (0.00-0.59)	0.27 (0.00-0.63)	0.634
A’D1.mean	4.49 (3.29-5.34)	5.04 (4.27-6.46)	<0.001	4.79 (4.34-6.15)	5.71 (4.82-7.13)	0.048
A’D1.sd	2.30 (1.85-2.67)	2.81 (2.31-3.35)	<0.001	2.85 (2.33-3.04)	3.10 (2.52-3.46)	0.018
A’D2.max	13.35 (10.57-15.53)	17.34 (15.47-19.98)	<0.001	15.18 (12.68-17.72)	18.80 (16.66-19.94)	<0.001
A’D2.min	0.00 (0.00-0.27)	0.00 (0.00-0.31)	0.233	0.02 (0.00-0.46)	0.12 (0.00-0.57)	0.648
A’D2.mean	4.48 (3.36-5.21)	5.12 (4.37-6.52)	<0.001	4.83 (4.17-5.83)	5.79 (5.06-6.96)	0.006
A’D2.sd	2.37 (1.87-2.74)	3.03 (2.42-3.48)	<0.001	2.77 (2.38-3.16)	3.27 (2.75-3.66)	0.003

Values are presented as mean ± SD, median (25th–75th percentiles) or n (%). P < 0.05 is regarded as statistically significant.

### 3.2 Selection of the optimal VP

**Figure 3:**
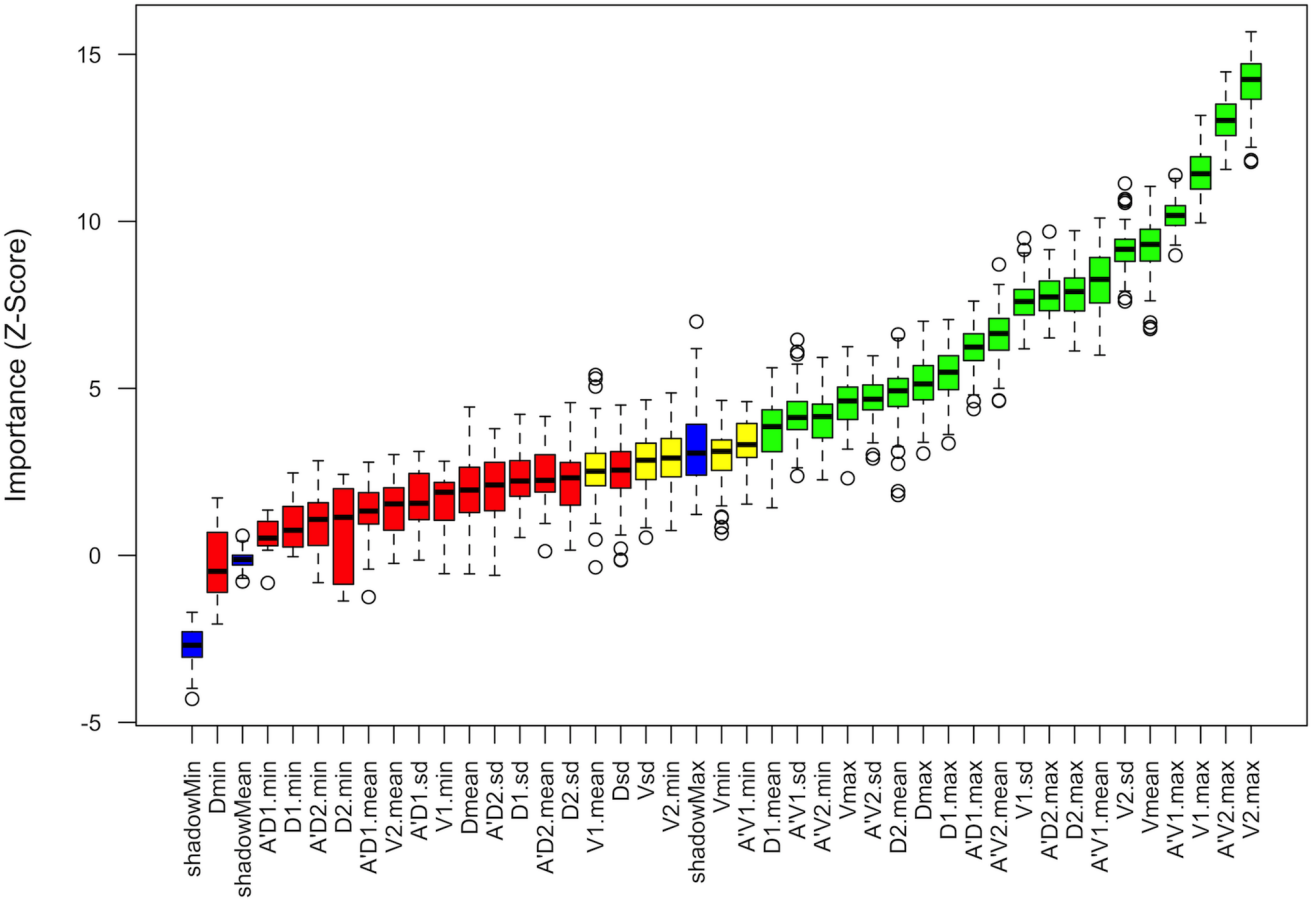
Feature selection using the Boruta algorithm. The boxplot displays the importance scores of the 40 viscous parameters (VPs) for differentiating benign from malignant breast lesions in the derivation cohort. V2.max (highlighted) demonstrated the highest importance score and was selected as the optimal parameter for subsequent model construction.

Except for A’V2.sd, nearly all VPs demonstrated strong multicollinearity (**Supplementary Table 1**), with A’D1.mean and A’D2.mean showing the most severe collinearity. Variable importance analysis based on the Boruta algorithm confirmed that 20 out of the initial 40 candidate VPs were valuable for differentiating benign from malignant breast lesions, including V2.max, A’V2.max, and V1.max (**Figure 3**). Consequently, V2.max (defined as the maximum viscosity value, in Pa·s, within the perilesional 2-mm rim based on the Voigt model), which had the highest importance score, was selected to construct the combined diagnostic model (BI-RADS-V) together with BI-RADS.

### 3.3 Predictive model development and performance evaluation

**Figure 4:**
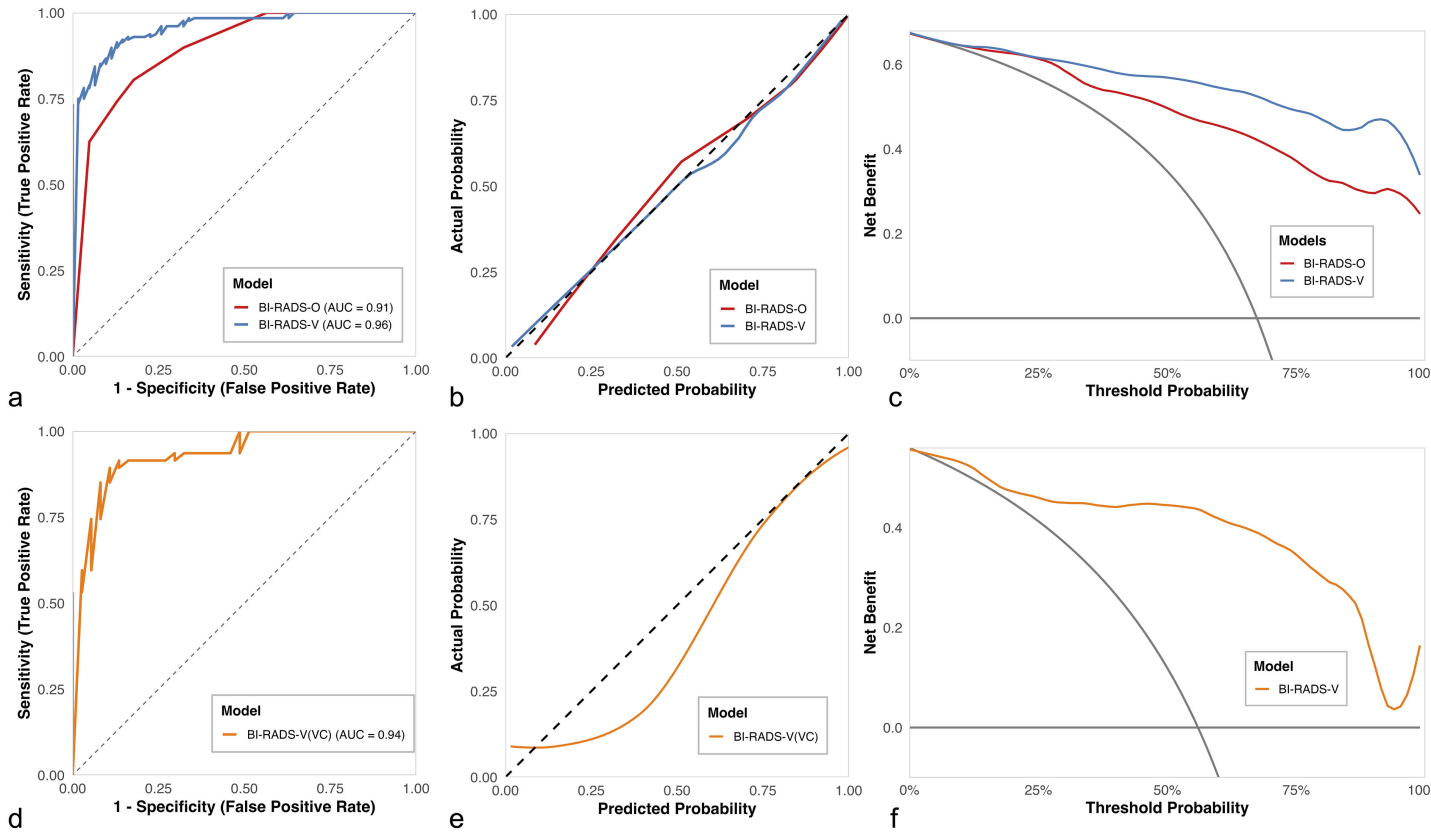
Performance comparison of the BI-RADS-V and BI-RADS-O models. **(a)** Receiver operating characteristic curves, **(b)** calibration curves, and **(c)** decision curve analysis for both models in the derivation cohort. **(d-f)** Corresponding validation results in the validation cohort. The combined model (BI-RADS-V) incorporating the viscous parameter V2.max showed superior discriminatory ability, calibration, and clinical utility compared to the model based on BI-RADS score alone (BI-RADS-O).

The performance metrics of the two models are summarized in **Table 3**. The results demonstrated that the BI-RADS-V demonstrated a significantly higher AUC (**Figure 4a**) of 0.96 (95% CI: 0.94–0.98) than the BI-RADS-O (AUC: 0.91, 95% CI: 0.87–0.95; DeLong’s test, *p* < 0.001). Furthermore, BI-RADS-V showed superior sensitivity (89.8%) and specificity (88.7%) compared with BI-RADS-O (sensitivity: 80.5%; specificity: 82.3%). The significant improvements were further quantified by the NRI (0.282, 95% CI: 0.130–0.432, *p* < 0.001) and IDI (0.179, 95% CI: 0.121–0.236, *p* < 0.001). The H-L test indicated no significant deviation between predicted and observed probabilities for either model (*p* > 0.05). The calibration curve (**Figure 4b**) for BI-RADS-V aligned more closely with the diagonal reference line and achieved a lower (superior) Brier score (0.076) compared to that of BI-RADS-O (0.116). The DCA (**Figure 4c**) showed that the net benefit curves of both models did not fall below the reference lines for “treat all” or “treat none” strategies. Notably, the curve for the BI-RADS-V consistently remained above that of the BI-RADS-O across a wide range of threshold probabilities. A sensitivity analysis of the BI-RADS-V model in the DC (**Supplementary Table 2**), conducted using Generalized Estimating Equation, confirmed its robustness, yielding nearly identical coefficient estimates with a negligible intraclass correlation (ICC = 0.041). In the VC, the BI-RADS-V demonstrated minimal performance degradation (**Table 3**). It achieved an AUC (**Figure 4d**) of 0.94 (95% CI: 0.89–0.99), a sensitivity of 91.5%, and a specificity of 83.8%. The H-L test result was non-significant (*p* = 0.170). The calibration curve showed excellent agreement with the diagonal reference line (**Figure 4e**), supported by a Brier score of 0.102. The DCA curve (**Figure 4f**) further demonstrated that the BI-RADS-V consistently remained above both reference lines. The complete specification of the logistic regression models, including regression coefficients, odds ratios, and the mathematical formula for calculating malignancy probability, is provided in **Supplementary Table 3**.

**Table 3:** Diagnostic performance of the predictive models.

Model	BI-RADS-O	BI-RADS-V	BI-RADS-V (VC)
AUC (95%CI)	0.91 (0.87 - 0.95)	0.96 (0.94 - 0.98)	0.94 (0.89-0.99)
Sensitivity (%)	80.5	89.8	91.5
Specificity (%)	82.3	88.7	83.8
Accuracy (%)	81.1	89.5	88.1
H-L test			
X^2^	10.248	1.434	11.597
p-value	0.115	0.963	0.170
Brier	0.116	0.076	0.102
NRI (95%CI)	0.282 (0.130-0.432)		\
NRI+ (95%CI)	0.023 (-0.041-0.089)		\
NRI- (95%CI)	0.258 (0.124-0.390)		\
IDI	0.179 (0.121 - 0.236)		\

BI-RADS-O, model based solely on the BI-RADS score; BI-RADS-V, combined model incorporating BI-RADS and the viscous parameter V2.max; BI-RADS-V(VC), performance of the BI-RADS-V model in the independent validation cohort. AUC, area under the ROC curve; NRI, net reclassification improvement; IDI, integrated discrimination improvement.

Analysis of subgroup performance demonstrated that the BI-RADS-V maintained high diagnostic efficacy (AUC > 0.90) across all predefined patient subgroups. Specifically, for the lesion size subgroup, the AUC was 0.93 (95% CI: 0.89–0.97) in lesions ≤20 mm (n = 114) and 0.99 (95% CI: 0.98–1.00) in lesions >20 mm (n = 76), a statistically significant difference (DeLong’s test, *p* = 0.007). Sensitivity (84.6% vs. 95.2%) and specificity (87.8% vs. 100%) were also superior in the large lesion group. In the age subgroup analysis, BI-RADS-V demonstrated AUC values of 0.98 (95% CI: 0.94–1.00) for those ≤45 years (n = 47) and 0.95 (95% CI: 0.92–0.98) for those >45 years (n = 143), with no statistically significant difference (DeLong’s test, *p* = 0.356). Sensitivity (90.9% vs. 92.5%) and specificity (96.0% vs. 86.5%) also showed comparable results between the two age groups.

### 3.4 Association between HER2 status and VPs:

As shown in **Table 4**, univariable analysis identified five VPs that differed significantly between HER2-positive and HER2-negative groups at the nominal level (p < 0.05), among which four (V1.max, V2.max, A’V1.max, and A’V2.max) showed significant associations with HER2 positivity. To account for multiple comparisons across the 40 VPs tested, a Bonferroni correction was applied (significance threshold set at p < 0.00125). After this stringent adjustment, only V2.max remained significantly associated with HER2 status (p < 0.001), demonstrating the strongest association with an odds ratio of 1.75 (95% CI: 1.31–2.50).

**Table 4:** Association between viscous parameters and HER2 status.

Parameter	Intergroup differences			Relevance		
	Negative (n=50)	Positive (n=39)	P value	OR	95%CI	P value
Voigt Parameters, Pa.s						
Vmax	4.46 (3.64, 5.48)	4.80 (3.50, 6.09)	0.376	1.16	(0.96, 1.45)	0.148
Vmin	0.00 (0.00, 0.00)	0.00 (0.00, 0.03)	0.026	12.93	(0.60, 3724.79)	0.219
Vmean	0.89 (0.61, 1.32)	0.88 (0.66, 1.38)	0.757	1.48	(0.82, 2.80)	0.205
Vsd	0.68 (0.47, 0.89)	0.66 (0.53, 0.85)	0.977	1.78	(0.61, 6.03)	0.306
V1.max	5.71 (4.53, 6.23)	6.74 (4.96, 8.55)	0.007	1.49	(1.17, 1.98)	0.003
V1.min	0.00 (0.00, 0.03)	0.00 (0.00, 0.06)	0.507	5.53	(0.79, 208.13)	0.188
V1.mean	1.26 (0.93, 1.56)	1.35 (0.93, 1.79)	0.614	1.35	(0.72, 2.62)	0.351
V1.sd	0.96 (0.78, 1.18)	1.09 (0.71, 1.36)	0.388	2.53	(0.81, 8.73)	0.122
V2.max	6.23 (5.32, 6.91)	7.45 (5.91, 9.04)	<0.001	1.75	(1.31, 2.50)	<0.001
V2.min	0.00 (0.00, 0.01)	0.00 (0.00, 0.03)	0.345	8.89	(0.39, 1446.04)	0.245
V2.mean	1.25 (0.95, 1.57)	1.38 (0.99, 1.73)	0.311	1.61	(0.81, 3.42)	0.187
V2.sd	0.99 (0.80, 1.21)	1.15 (0.79, 1.38)	0.299	2.98	(0.86, 11.54)	0.096
A’V1.max	5.83 (4.70, 6.41)	6.07 (4.83, 8.60)	0.032	1.37	(1.10, 1.76)	0.009
A’V1.min	0.00 (0.00, 0.00)	0.00 (0.00, 0.01)	0.089	7.48	(0.42, 1321.59)	0.279
A’V1.mean	1.00 (0.70, 1.41)	1.01 (0.72, 1.46)	0.735	1.48	(0.81, 2.85)	0.209
A’V1.sd	0.80 (0.59, 0.95)	0.85 (0.57, 1.02)	0.682	2.12	(0.71, 7.37)	0.197
A’V2.max	6.29 (5.32, 6.94)	7.60 (5.91, 9.04)	0.002	1.64	(1.25, 2.28)	0.001
A’V2.min	0.00 (0.00, 0.00)	0.00 (0.00, 0.01)	0.113	11.35	(0.40, 3781.56)	0.243
A’V2.mean	1.09 (0.80, 1.43)	1.09 (0.81, 1.52)	0.629	1.55	(0.82, 3.09)	0.184
A’V2.sd	0.85 (0.68, 1.02)	0.93 (0.65, 1.12)	0.454	2.81	(0.87, 10.91)	0.102
SWD Parameters, m/s/kHz						
Dmax	16.43 (15.00, 19.20)	16.02 (14.32, 19.21)	0.476	0.92	(0.80, 1.07)	0.283
Dmin	0.02 (0.00, 0.63)	0.26 (0.00, 0.77)	0.230	1.29	(0.62, 2.79)	0.501
Dmean	5.20 (4.28, 6.51)	4.83 (3.87, 6.10)	0.199	0.88	(0.69, 1.11)	0.289
Dsd	2.90 (2.34, 3.24)	2.74 (2.04, 3.01)	0.261	0.83	(0.51, 1.30)	0.424
D1.max	16.76 (15.45, 19.43)	16.39 (15.50, 19.60)	0.661	0.94	(0.80, 1.09)	0.413
D1.min	0.15 (0.00, 0.84)	0.21 (0.00, 0.93)	0.805	0.96	(0.51, 1.78)	0.899
D1.mean	5.94 (4.96, 7.31)	5.54 (4.96, 7.08)	0.406	0.88	(0.66, 1.14)	0.333
D1.sd	3.36 (2.92, 3.71)	3.23 (2.73, 3.74)	0.716	0.9	(0.55, 1.42)	0.643
D2.max	18.10 (15.72, 19.99)	17.40 (16.12, 19.96)	0.593	0.92	(0.78, 1.08)	0.328
D2.min	0.01 (0.00, 0.65)	0.03 (0.00, 0.36)	0.727	0.61	(0.25, 1.29)	0.223
D2.mean	5.73 (5.05, 7.03)	5.49 (4.91, 6.89)	0.439	0.88	(0.66, 1.17)	0.389
D2.sd	3.39 (2.96, 3.74)	3.22 (2.98, 3.78)	0.713	0.94	(0.57, 1.52)	0.793
A’D1.max	17.30 (16.03, 19.99)	17.13 (15.86, 19.98)	0.668	0.92	(0.78, 1.08)	0.324
A’D1.min	0.01 (0.00, 0.34)	0.06 (0.00, 0.71)	0.375	1.25	(0.56, 2.87)	0.575
A’D1.mean	5.46 (4.44, 6.76)	5.00 (4.09, 6.19)	0.231	0.87	(0.68, 1.11)	0.280
A’D1.sd	3.02 (2.52, 3.33)	2.83 (2.34, 3.23)	0.350	0.84	(0.51, 1.34)	0.473
A’D2.max	18.16 (16.06, 20.00)	17.86 (16.12, 19.98)	0.635	0.91	(0.76, 1.08)	0.281
A’D2.min	0.00 (0.00, 0.29)	0.03 (0.00, 0.36)	0.622	0.87	(0.34, 2.03)	0.746
A’D2.mean	5.53 (4.49, 6.77)	5.14 (4.27, 6.38)	0.263	0.87	(0.66, 1.12)	0.288
A’D2.sd	3.10 (2.71, 3.43)	2.92 (2.54, 3.43)	0.462	0.87	(0.52, 1.42)	0.576

Parameters are presented as median (interquartile range). Intergroup differences were analyzed using the Mann-Whitney U test. Associations with HER2 positivity were assessed by univariable logistic regression and expressed as odds ratios (ORs) with 95% confidence intervals (CIs). A Bonferroni-corrected significance threshold of p < 0.00125 was applied to account for multiple comparisons across 40 parameters.

### 3.5 Intra-observer reproducibility

The intra-observer reproducibility analysis for the pivotal viscous parameter, V2.max, demonstrated excellent reliability. The ICC for absolute agreement was 0.908 (95%CI: 0.889–0.925), indicating a high degree of measurement consistency for this parameter when assessed by a single experienced sonographer.

## 4 Discussion

The findings of this study lead to two principal conclusions: (1) The integration of BI-RADS with the optimal VP (V2.max), is associated with enhanced differentiation between benign and malignant breast lesions while demonstrating excellent generalizability. (2) To our knowledge, this is among the first studies to suggest a correlation between VPs and HER2 status and to explore potential explanations for this association.

Importance scoring using the Boruta algorithm demonstrated that VPs derived from the Voigt model were ranked higher than those based on the SWD model, with 13 out of 20 parameters classified as “important” originating from the Voigt model. This discrepancy may be attributed to the complex Voigt model’s superior ability to characterize tissue viscosity compared to the linear-fitting-based SWD model (12), a conclusion that corroborates the work of Jia et al. (14). A key distinction, however, lies in the specific optimal parameter selected: our study identified V2.max as the VP best representing UVI, whereas Jia et al. employed A’V2.max. The observed discrepancy may be explained by the “stiff rim sign” in shear wave elastography of malignant breast lesions. This sign pathologically reflects altered mechanical properties in the perilesional area, primarily due to connective tissue hyperplasia and tumor cell infiltration, which consequently lead to elevated shear wave velocity (24). Conversely, the lesion core often exhibits lower shear wave velocity, likely attributable to increased wave attenuation (25, 26). The inferior performance of A’V2.max could be due to its compositional nature. By integrating viscosity features from both the lesion and surrounding tissue, its value was likely confounded by the low shear wave velocity within the lesion core, ultimately diminishing its diagnostic utility compared to the more specific V2.max. Differences in screening strategies may also explain the variation in results. To directly address the inherent multicollinearity among the VPs, as demonstrated by our variance inflation factor analysis, we employed the Boruta algorithm. Unlike binary logistic regression, which is ill-equipped to handle complex variable interactions and multicollinearity, the Boruta algorithm effectively manages these challenges, thereby yielding a more robust and reliable variable selection. Our analysis further affirms the technical robustness of UVI, demonstrating excellent intra-observer reproducibility for the key parameter V2.max (ICC = 0.908). This indicates that a trained operator can obtain highly consistent measurements, a crucial prerequisite for the clinical translation of this quantitative biomarker.

Integration of the optimal VP (V2.max) with the BI-RADS into a logistic regression model yielded the BI-RADS-V, which was significantly more accurate than the original BI-RADS-O (AUC: 0.96 vs. 0.91; *p* < 0.001). Importantly, BI-RADS-V not only increased sensitivity (89.8% vs. 80.5%) but also provided a critical gain in specificity (88.7% vs. 82.3%), thereby mitigating the well-documented limitation of low specificity in conventional ultrasound for breast lesion diagnosis (27). The significant NRI (0.282) and IDI (0.179) further validated the diagnostic advantage of the BI-RADS-V. Further evaluation of model calibration confirmed the superiority of BI-RADS-V over BI-RADS-O. The calibration curve for BI-RADS-V aligned more closely with the diagonal reference line, supported by a more favorable H-L test result (*p*: 0.963 vs. 0.115) and a lower (superior) Brier score (0.076 vs. 0.116). Collectively, these metrics affirm the greater reliability of the BI-RADS-V. Furthermore, DCA revealed that the BI-RADS-V yielded a consistently greater net benefit than the BI-RADS-O over a broad spectrum of clinically relevant threshold probabilities. This finding underscores the superior clinical utility of the BI-RADS-V. The BI-RADS-V also demonstrated strong generalizability to the VC. This was evidenced by a stable AUC (0.96 in DC vs. 0.94 in VC), along with robust sensitivity (91.5%) and specificity (83.8%). Its reliable performance was further supported by a calibration curve closely aligned with the diagonal, a non-significant H-L test (*p* = 0.170), and a low Brier score (0.102). The superior performance of the BI-RADS-V may be explained by the increased viscous properties observed in malignant breast lesions, resulting from biomechanical changes in cellular and extracellular matrix architecture (28, 29). Compared to BI-RADS-O, BI-RADS-V incorporated additional analysis of the viscous properties, enabling a more comprehensive evaluation of breast lesions. Furthermore, the introduction of VP as an objective quantitative metric contributed to enhanced diagnostic stability. Although our findings align with those of Jia et al. (14), a key methodological distinction exists: rather than merely applying an optimal VP cut-off value as an additional criterion for upgrading BI-RADS categories, our study developed an interpretable binary logistic regression model. It is undeniable that the study by Jia et al. represents a pioneering effort in the clinical application of breast UVI, particularly due to its simplicity. Their method, which requires no complex calculations and adjusts the BI-RADS categories based solely on a cut-off value, follows an analytical paradigm that integrates well with sonographers’ established workflows, facilitating smoother clinical adoption. However, an important limitation of this approach is that rule-based upgrades of BI-RADS categories and the dichotomization of the continuous VP into a binary variable may lead to loss of statistical information. To address this, we constructed a diagnostic model that both improves interpretability and fully utilizes the information contained in the VP, thereby offering a stronger foundation for advancing UVI applications.

To further assess the BI-RADS-V, we evaluated its diagnostic performance across different patient subgroups. Although no statistically significant difference in performance was observed between age groups (*p* = 0.356), BI-RADS-V achieved a higher AUC in the younger patient cohort (0.98 vs. 0.95). The underlying mechanism may involve the higher prevalence of the aggressive triple-negative BC subtype in younger patients (23). Triple-negative BC is characterized by a highly fibrotic stromal microenvironment (30), which is known to confer elevated tissue viscosity (28). This increased viscosity likely creates a more discernible contrast with the viscosity of benign lesions, facilitating identification by UVI. A significant performance difference was observed based on lesion size, with BI-RADS-V demonstrating superior diagnostic accuracy for larger lesions (AUC: 0.99 vs. 0.93, *p* = 0.007). We hypothesize that the mechanism underlying this divergence is the more advanced stromal remodeling and fibrosis typically present in larger lesions (31, 32), which confer greater viscous properties. The subgroup analysis confirms the high diagnostic performance of BI-RADS-V in both age and lesion size subgroups, while highlighting its optimal performance in younger patients and those with large lesions. This demonstrates the model’s robustness and, more importantly, suggests that it holds particular promise for application in these specific demographic and clinical contexts. Taken together with its previously demonstrated strong diagnostic performance, these findings support the notion that UVI holds considerable promise for discriminating between benign and malignant breast lesions.

HER2 is an important therapeutic target in breast cancer, with its overexpression strongly linked to increased tumor aggressiveness and poorer prognosis (33). Although targeted therapies against HER2 have significantly improved patient survival, the current standard methods for its detection—IHC and FISH—are invasive, costly, and impractical for repeated monitoring. This inherent limitation underscores the pressing clinical need for non-invasive techniques to assess HER2 status. In this context, our study provides preliminary evidence that UVI may offer a novel solution. Notably, our analysis indicated a notable correlation (*p* < 0.05) between HER2 status and four Voigt model-derived VPs, with V2.max showing the strongest association (odds ratio = 1.75). This phenomenon may be related to HER2-mediated hypoxia (34), a condition that a condition that is known to promote collagen production in the extracellular matrix (35) and may consequently lead to elevated viscosity (28). Importantly, we observed that the HER2-associated VPs were specifically linked to the viscosity properties of the perilesional stroma. This is supported by Gan et al. (30), who reported that stromal changes in HER2-positive cancers (including collagen proliferation) are spatially heterogeneous, being most pronounced at the invasive front and around tumor nests. Thus, we hypothesize that UVI has the potential to contribute to the non-invasive assessment of HER2 status by quantifying characteristic peritumoral viscosity alterations resulting from non-uniform connective tissue proliferation. It should be noted, however, that Gan et al.’s work focused solely on cellular histology and did not control for estrogen receptor or progesterone receptor expression levels. Therefore, although we provide a plausible interpretation for the observed correlation, the proposed histological link must be viewed as a hypothesis requiring further validation.

This study has several limitations. First, its single-center, retrospective design and the enrollment of patients scheduled for biopsy or surgery (“suspected malignant” cases) may introduce selection bias and limit the generalizability of our findings to a true screening population. Second, the statistical analysis was performed at the lesion level, and the potential lack of independence for the few patients with multiple lesions was not accounted for, which might have influenced the results. Third, regarding the reproducibility of UVI measurements, while excellent intra-observer reliability was confirmed, the inter-observer reproducibility across different operators was not assessed. Fourth, we focused on the combined model’s value and did not evaluate the discriminatory power of the VP alone. Finally, the exploratory analysis of HER2 status, while hypothesis-generating, was not adjusted for multiple comparisons or other clinicopathological factors, and the underlying histological basis for the observed correlation remains unclear. Therefore, future large-scale, multicenter studies that include diverse clinical populations and molecular subtypes are essential to advance the field of UVI for breast lesions.

In summary, the integration of UVI with the BI-RADS system was associated with improved diagnostic accuracy and showed promising generalizability in our study. Furthermore, the observed association between perilesional viscous features and HER2 status suggests its potential as a non-invasive indicator of molecular subtypes. These findings support continued investigation of UVI in the personalized evaluation of breast lesions.

## Data Availability

The raw data supporting the conclusions of this article will be made available by the authors, without undue reservation.
