# Supplementary material for: Ultrasound viscosity imaging empowers BI-RADS: toward precise breast lesion diagnosis and analysis of HER2 status
Source: Front Oncol. 2026 Feb 25;16:1726418. doi: 10.3389/fonc.2026.1726418 (PMC12975447; doi:10.3389/fonc.2026.1726418)
Supplement: Supplementary file 1 [file Table1.docx]

**Supplementary Table S1.** **Variance Inflation Factors for the Vascular Parameters**

| **Voigt Model** | | | | **SWD Model** | | | | |
| --- | --- | --- | --- | --- | --- | --- | --- | --- |
| Max | Min | Mean | Sd | | Max | Min | Mean | Sd |
| Vmax  (12.82 ) | Vmin  (13.89) | Vmean  (272.66) | Vsd  (72.92) | | Dmax  (18.48) | Dmin  (12.83) | Dmean  (699.33) | Dsd  (213.89) |
| V1.max  (188.23) | V1.min  (9.99) | V1.mean  (87.53) | V1.sd  (73.58) | | D1.max  (78.03) | D1.min  (13.22) | D1.mean  (436.87) | D1.sd  (240.33) |
| V2.max  (301.39) | V2.min  (21.11) | V2.mean  (98.32) | V2.sd  (37.48) | | D2.max  (96.06) | D2.min  (19.00) | D2.mean  (517.80) | D2.sd  (271.07) |
| A'V1.max  (200.40) | A'V1.min  (81.96) | A'V1.mean  (633.26) | A'V1.sd  (160.30) | | A'D1.max  (120.53) | A'D1.min  (38.59) | A'D1.mean  (4374.66) | A'D1.sd  (1338.05) |
| A'V2.max  (310.72) | A'V2.min  (90.01) | A'V2.mean  (303.04) | A'V2.sd  (3.93) | | A'D2.max  (126.61) | A'D2.min  (27.66) | A'D2.mean  (2075.85) | A'D2.sd  (747.74) |

Note: A VIF value > 5 indicates a concerning degree of multicollinearity, and a value > 10 indicates severe multicollinearity.​​ As demonstrated, all VPs exhibited strong multicollinearity (VIF > 10) except for A'V2.sd. The parameters A'D1.mean and A'D2.mean showed the highest VIF values, indicating the most severe collinearity. VIF, Variance Inflation Factor; VP, Vascular Parameter.​​
