# Supplementary material for: Ultrasound viscosity imaging empowers BI-RADS: toward precise breast lesion diagnosis and analysis of HER2 status
Source: Front Oncol. 2026 Feb 25;16:1726418. doi: 10.3389/fonc.2026.1726418 (PMC12975447; doi:10.3389/fonc.2026.1726418)
Supplement: Supplementary file 2 [file Table2.docx]

**Supplementary Table S2. Sensitivity Analysis for Clustering Effects on the BI-RADS-V Model in the Derivation Cohort**

| Variable | Logistic Regression Model | GEE Model | Difference |
| --- | --- | --- | --- |
| **Coefficient (Std. Error)**​ | | | |
| Intercept | -9.679 (1.521) | -9.669 (1.291) | +0.010 |
| BI-RADS | 0.727 (0.146) | 0.726 (0.121) | -0.001 |
| V2.max | 0.799 (0.161) | 0.799 (0.149) | 0.000 |
| **Model Performance** | | | |
| AUC（95%CI） | 0.959 | 0.959 | Identical |
| Residual Deviance | 91.107 |  |  |
| **Clustering Parameters​** | | | |
| Number of Clusters |  | 174 |  |
| Number of Clusters |  | 3 |  |
| Intraclass Correlation Coefficient (α) |  | 0.041 (SE=0.362) |  |

Note: The GEE model employed an exchangeable correlation structure. An α value of 0.041 indicates a very weak clustering effect. GEE, Generalized Estimating Equation.SE, Standard Deviation.
