# Supplementary material for: Ultrasound viscosity imaging empowers BI-RADS: toward precise breast lesion diagnosis and analysis of HER2 status
Source: Front Oncol. 2026 Feb 25;16:1726418. doi: 10.3389/fonc.2026.1726418 (PMC12975447; doi:10.3389/fonc.2026.1726418)
Supplement: Supplementary file 3 [file Table3.docx]

**Supplementary Table S3. Coefficients, Odds Ratios, and Confidence Intervals for the Diagnostic Models**

| Model | Parameter | Estimate (β) | Std. Error | OR (95% CI) | p-value |
| --- | --- | --- | --- | --- | --- |
| BI-RADS-O | Intercept | -6.392 | 0.999 | 0.002 (0.0002, 0.010) | < 0.001 |
|  | BI-RADS | 0.805 | 0.120 | 2.236 (1.81, 2.90) | < 0.001 |
| **BI-RADS-V​** | Intercept | -9.679 | 1.521 | < 0.001 (0.000002, 0.001) | < 0.001 |
|  | BI-RADS | 0.727 | 0.146 | 2.070 (1.60, 2.86) | < 0.001 |
|  | V2.max | 0.799 | 0.161 | 2.223 (1.67, 3.16) | < 0.001 |

Note: OR, Odds Ratio.
